# Efficacy and safety of nivolumab plus ipilimumab in gastrointestinal cancers: a systematic review and meta-analysis

**DOI:** 10.3389/fonc.2024.1515992

**Published:** 2025-01-07

**Authors:** Bowen Dai, Jiaping Jiang, Xiaoyu Yu, Haihua Zhan, Zhengchuan Hu

**Affiliations:** ^1^ Southwest Medical University, Luzhou, China; ^2^ Department of General Surgery (Gastrointestinal Surgery), The Affiliated Hospital of Southwest Medical University, Luzhou, China

**Keywords:** nivolumab, ipilimumab, gastrointestinal cancer, objective response rate, efficacy, meta-analysis

## Abstract

**Introduction:**

Gastrointestinal (GI) cancers represent a significant global health burden, and the need for more effective treatment options is exceptionally pressing. The present meta-analysis aimed to explore the efficacy and safety of the combination of nivolumab and ipilimumab in treating GI cancers.

**Methods:**

A systematic search of four databases (PubMed, Embase, Web of Science, and Cochrane Library) was conducted for articles on the treatment of GI cancers with nivolumab combined with ipilimumab, published from 2014 up to 30 August 2024. The inclusion criteria were designed according to the principles of Participants, Intervention, Control, Outcomes, and Study (PICOS). The control group was chemotherapy or nivolumab monotherapy or nivolumab in combination with other drugs. We extracted data from 10 randomized controlled trials and utilized a random effects model to assess the objective response rate (ORR), median progression-free survival (mPFS), median overall survival (mOS), median duration of response (mDOR), and treatment-related adverse events (TRAEs). The data analysis was conducted using Review Manager version 5.4 and Stata version 12.0.

**Results:**

Overall, the combination of nivolumab and ipilimumab demonstrated superior outcomes, including a higher ORR (OR = 1.69, P = 0.01), prolonged mOS (MD = 1.74, P = 0.04) and extended mDOR (MD = 5.64, P < 0.00001) compared to the control group. Subgroup analysis demonstrated that the ORR (OR = 1.75, P = 0.02) and mOS (MD = 5.02, P = 0.003) were significantly improved in patients with esophageal cancer. Notably, the ORR in patients with biliary cancer was significantly lower (OR = 0.11, P = 0.04). Additionally, the ORR was significantly higher in the NIVO1 + IPI3group (OR = 2.82, P = 0.01) and NIVO3 + IPI1 group (OR = 1.62, P = 0.01). Regarding safety, there was no statistically significant difference between the combination regimen and the control group in terms of any grade (OR = 0.72, P = 0.26) or grade 3-4 TRAEs (OR = 1.36, P = 0.14).

**Conclusions:**

Nivolumab in combination with ipilimumab demonstrated significant efficacy in GI cancers (especially esophageal cancer) without causing more adverse reactions. However, its efficacy in biliary cancer still needs to be further proven.

**Systematic review registration:**

https://www.crd.york.ac.uk/prospero/, identifier CRD42024590994.

## 1 Introduction

Today, the burden of cancer is one of the world’s greatest public health problems (1). Gastrointestinal (GI) cancers constitute a significant category of neoplasms, encompassing a range of digestive tract tumors, including those affecting the colon, rectum, esophagus, stomach, liver, pancreas, gallbladder, and bile ducts. These cancers represent a significant global health burden, with a prevalence rate exceeding 26% and a mortality rate exceeding 35% (2). Immunotherapy, particularly immune checkpoint blockade and targeting the tumor immune microenvironment, has been extensively employed in the treatment of numerous GI cancers, including microsatellite instability-high (MSI-H) colorectal cancer, gastric cancer, and hepatocellular carcinoma (3–5). While immunotherapy has demonstrated considerable efficacy in the treatment of numerous tumors, it can also induce adverse events related to the immune system, particularly in the case of immune checkpoint inhibitors (6, 7). Accordingly, there is a clear need to investigate the development of more efficacious and safer immune checkpoint target drugs.

Currently, data from several clinical trials show satisfactory therapeutic effects of immune checkpoint inhibitors in patients with GI cancers, such as HER-2, PD-1/PD-L1, and CTLA4-targeted therapy (8–10). Cytotoxic T-lymphocyte-associated antigen 4 (CTLA4, also designated as CD152) and programmed cell death protein 1 (PD1, also designated as CD279) represented two of the most intensively investigated targets in the domain of clinical immunotherapy. CTLA4 is an immune checkpoint receptor that is predominantly expressed in T cells. It has the same receptor as CD28 but exhibits a higher overall affinity. By inhibiting the CTLA-4 receptor-ligand interaction through the use of an anti-CTLA-4 antibody, the CD28-mediated T cell stimulation signal is enhanced, thereby achieving an anti-tumor immune effect (11, 12). PD-1 is expressed at a greater level than CTLA4 in activated T cells, B cells, and myeloid cells. It inhibits T cell activation, affects the tumor microenvironment and tolerance, and so forth, by interacting with two ligands, PD-L1 and PD-L2, which partially overlap in their functions (13–15). There is mounting evidence that targeting the PD-1/PD-L pathway represents an efficacious treatment strategy for augmenting anti-tumor immune responses. Antibody-mediated PD-1 or PD-L1 blockade held immense clinical promise for a range of advanced tumors (including non-small cell lung cancer, melanoma, gastroesophageal cancer, hepatocellular carcinoma, and others) in comparison to chemotherapy or palliative care (16–19). Concurrently, research has demonstrated that the combination of anti-CTLA-4 and anti-PD-1 therapies is regarded as a more efficacious approach (20). The simultaneous blockade of these two molecules may result in a synergistic effect, whereby they act on CD28 and participate in signal pathways such as T cell activation, thereby enhancing T cell activity. However, the relative contribution of the various known molecular mechanisms of CTLA4 and PD-1 blockade to the therapeutic effect remains to be further explored (21, 22). Nivolumab and ipilimumab are monoclonal antibodies that target PD-1 and CTLA-4, respectively. The combination of anti-PD-1 and anti-CTLA-4 therapy (nivo-ipi) has been officially approved by the US Food and Drug Administration (FDA) for the treatment of a variety of cancers, including colorectal cancer, hepatocellular carcinoma, and other cancers (23, 24). Therefore, the combination of nivolumab and ipilimumab is expected to become a new and more effective treatment option for GI cancers.

Some studies have demonstrated that the combination of nivolumab and ipilimumab yielded promising clinical outcomes in the treatment of GI cancers (including gastroesophageal cancer, esophageal squamous cell carcinoma, pancreatic cancer, and hepatocellular carcinoma) (10, 25–28). Nevertheless, the advantages of this therapeutic approach in the context of other GI cancers (e.g., colorectal and biliary cancers) remain a matter of contention, and the overall efficacy and safety of this regimen in GI cancers has yet to be fully evaluated (29). Although some meta-analyses indicated that the combination of nivolumab and ipilimumab may be an effective treatment for second-line therapy of advanced hepatocellular carcinoma and the third-line treatment of advanced gastric cancer, the current evidence was insufficient to conclude the efficacy of this regimen for all GI cancers (30, 31). Moreover, there is a dearth of direct efficacy assessments of different tumor types and different dose ratios of the combination, which is essential to demonstrate the universality and heterogeneity of treatment options.

In order to ascertain the overall efficacy and safety of this combination in the treatment of GI cancers, as well as variations in efficacy across tumor types and dose ratios, and to enhance the clinical feasibility of this combination in the treatment of GI cancers, we conducted a meta-analysis. The results of the analysis may contribute to the development of clinical decision-making and provide potential new options for the first-line treatment of GI cancers. This will facilitate the development of optimal treatment strategies for future patients undergoing treatment with GI tumors.

## 2 Method

### 2.1 Search strategy

This systematic review and meta-analysis followed the PRISMA statement and was registered with PROSPERO (CRD42024590994). A systematic search was conducted in four databases, namely PubMed, Embase, Web of Science, and Cochrane Library to identify relevant articles from 2014 to August 30, 2024, using the following keywords: (Ipilimumab) and (Nivolumab) and (Stomach Neoplasms or Esophageal Neoplasms or Liver Neoplasms or Colonic Neoplasms or Rectal Neoplasms or Colorectal Neoplasms or Pancreatic Neoplasms or Gallbladder Neoplasms or Bile Duct Neoplasms or Gastrointestinal Neoplasms). In addition, we searched the gray literature. The search strategy was constructed following the PICOS framework and comprised the integration of both Medical Subject Headings (MeSH) terms and free-text keywords. For articles with missing or incomplete data, we contacted the authors by email to obtain complete data. Additionally, we sought relevant literature that is not readily available through standard sources by reaching out to subject matter experts in the field.

### 2.2 Inclusion and exclusion criteria

Inclusion criteria were as follows: (a) patients diagnosed with all GI cancer types; (b) the combination therapy of nivolumab and ipilimumab was used as the experimental group; (c) chemotherapy or monotherapy with nivolumab or combination therapy with nivolumab and other drugs was used as the control group; (d) at least one of the following outcomes was reported: overall survival (OS), progression-free survival (PFS), objective response rate (ORR), duration of response (DOR), and treatment-related adverse events (TRAEs).

Additionally, studies that met the following criteria were excluded: (a) other types of articles, such as observational study designs (retrospective/prospective), single-arm design studies, case reports, publications, animal studies, and conference proceedings; (b) duplicate patient cohorts; (c) cancers that are not GI cancers; (d) other unrelated researches.

### 2.3 Study selection

All literature was imported into EndNote (Version 20; Clarivate Analytics) and deduplicated using a combination of automatic and manual methods. Subsequently, two reviewers (Bowen Dai, Haihua Zhan) independently screened the titles and abstracts of retrieved articles. Any disagreements were resolved through discussion. In cases of disagreement, a third reviewer took on the role of a mediator.

### 2.4 Quality assessment

Two authors (Haihua Zhan and Xiaoyu Yu) undertook an independent assessment of the risk of bias of each included randomized controlled trial using the Cochrane Risk of Bias Assessment Tool, which assesses six dimensions: random sequence generation, allocation concealment, blinding of participants and personnel, blinding of outcome assessment, incomplete outcome data, and selective reporting (32). Three levels are defined: ‘unclear risk’, ‘low risk’, and ‘high risk’. Any disagreements between authors were resolved by discussion with an independent third author.

### 2.5 Data extraction

The data were extracted by two independent reviewers (Bowen Dai, Jiaping Jiang) and included the following items: the first author, study type, number of participants, sex ratio, median age, primary endpoint, and treatment experimental arm. The following outcome indicators are more appropriate: mOS, mPFS, ORR, mDOR, and TRAEs. To ensure data accuracy, two authors independently performed data extraction. Any discrepancies were resolved through consensus discussions.

### 2.6 Statistical analysis

Statistical analysis was performed using the Cochrane Review Manager (Review Manager Version 5.4) and Stata 12.0 software. The effect size was calculated using a standardized mean difference, and a 95% confidence interval (CI) was generated. Given that the studies included in the analysis originate from the public literature, it would be more reasonable to select the random effect model as the preliminary model. Furthermore, it was determined that a p-value less than 0.05 would signify statistical significance.

The primary endpoints of this study were mOS, mPFS. Secondary endpoints were ORR, mDOR, and TRAEs. Given the heterogeneity, all pooled analyses were performed using a random effects model. The Cochran Q statistic and the I² statistic were employed to assess the presence of heterogeneity. If the Q statistic yielded a statistically significant result (P < 0.05), the I² statistic quantified the proportion of sample differences attributable to heterogeneity. I^2^ values exceeding 25%, 50%, and 75% were considered to be low, medium, and high heterogeneity, respectively (33). Given the considerable heterogeneity observed in mOS and mPFS, we performed a subgroup analysis to investigate potential differences in efficacy across different tumor types. In addition, we conducted a sensitivity analysis to ascertain whether the exclusion of studies exhibiting aberrant characteristics could account for the observed heterogeneity and influence the pooled effect.

## 3 Results

**Figure 1:**
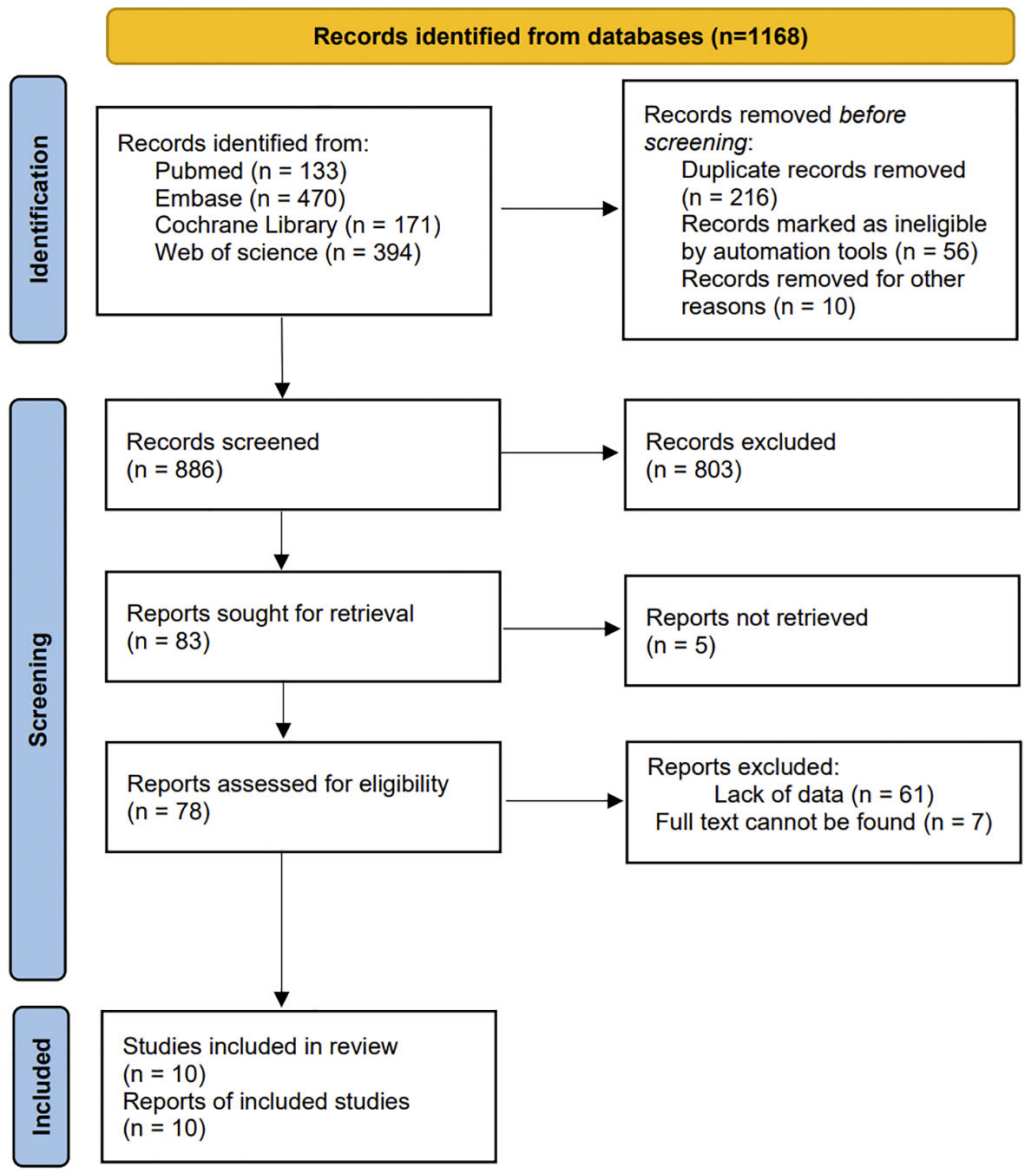
Literature screening process.

A total of 1168 published studies were identified through an initial database search. After removing 282 studies using EndNote, the remaining 886 studies were screened. By reading the title and abstract, we excluded 803 unrelated articles. The remaining 83 articles then underwent full-text searching and reading. Of these, 5 could not retrieved, 61 were found to lack data, and 7 could not be located in their entirety. Consequently, a total of 10 studies were ultimately included for data extraction. The complete screening process is illustrated in **Figure 1**.

### 3.1 Characteristics of the included studies

**Table 1** provides details of each study. In total, 10 trials involving 3056 patients met predefined inclusion criteria. All studies included in this systematic review and meta-analysis were randomized controlled trials (10, 25–29, 34–37). The average age of the included samples was 63.1 years old, with the majority of men (79.08%). One of the studies did not provide information on the age and gender of the population due to differences in its main experimental plan (34). The 10 primary endpoints of the trials include PFS, OS, DOR, ORR, dose-limiting toxicity (DLT), safety, and tolerability. We extracted the ORR, mOS, mPFS, mDOR, and TRAEs reported in the literature for summary analysis.

**Table 1:** Characteristics of studies included in meta-analysis.

Study	Author	Study type	Participants No.	Males (%)	Median age	Primary endpoint	Treatment Experimental Arm
1	Chen 2022	RCT	84	52.4	64.5	CBR	nivolumab plus ipilimumab plus SBRT or nivolumab plus gemcitabine plus cisplatin plus SBRT
2	Elez 2024	RCT	75	64	60.3	DLT ORR	nivolumab plus ipilimumab plus binimetinib or nivolumab plus binimetinib
3	Janjigian 2018	RCT	160	77.5	57.2	ORR	nivolumab plus ipilimumab or nivolumab
4	Juloori 2023	RCT	13	85	67	DLT	nivolumab plus ipilimumab plus SBRT or nivolumab plus gemcitabine plus cisplatin plus SBRT
5	Kaseb 2022	RCT	27	70	63.1	safety tolerability	nivolumab plus ipilimumab or nivolumab
6	Kato 2023	RCT	268	86.6	66.5	OS PFS	nivolumab plus ipilimumab or chemotherapy
7	Kato 2024	RCT	649	83.8	63	OS PFS	nivolumab plus ipilimumab or chemotherapy
8	Sahai 2022	RCT	68	51.4	62.5	PFS	nivolumab plus ipilimumab or nivolumab plus gemcitabine plus cisplatin
9	Shitara 2022	RCT	1641	none	none	OS PFS	nivolumab plus ipilimumab or chemotherapy
10	Yau 2023	RCT	71	87.3	65.7	safety tolerability ORR DOR	nivolumab plus ipilimumab plus cabozantinib or nivolumab plus cabozantinib

CBR, clinical benefit rate; ORR, objective response rate; DLT, dose-limiting toxicity; OS, overall survival; PFS, progression-free survival; DOR, duration of response.

Of the 10 RCTs included, 3 trials compared the efficacy of nivolumab plus ipilimumab with chemotherapy (10, 34, 35). 2 trials compared the efficacy of nivolumab plus ipilimumab with nivolumab (26, 36). 1 trial compared the efficacy of nivolumab plus ipilimumab plus binimetinib with nivolumab plus binimetinib (37). 1 trial compared the efficacy of nivolumab plus ipilimumab plus cabozantinib with nivolumab plus cabozantinib (27). 2 trials compared the efficacy of nivolumab plus ipilimumab plus stereotactic body radiotherapy (SBRT) with nivolumab plus gemcitabine plus cisplatin plus SBRT (25, 28). 1 trial compared the efficacy of nivolumab plus ipilimumab with nivolumab plus gemcitabine plus cisplatin (29).

### 3.2 Quality assessment and publication bias

The risk of bias was discussed and assessed according to the Cochrane Collaboration’s Risk of Bias tool by two independent investigators (Haihua Zhan, Xiaoyu Yu), the risk of bias of the included literature was assessed in terms of the following six dimensions: random sequence generation (selection bias), allocation concealment (selection bias), blinding of participants and personnel (performance bias), blinding of outcome assessment (detection bias), incomplete outcome data (attrition bias), and selective reporting (reporting bias), and was categorized into three types: low risk, high risk, and uncertain risk. Data extraction was conducted by mutual agreement and all potential disagreements were resolved by consensus.

**Figure 2:**
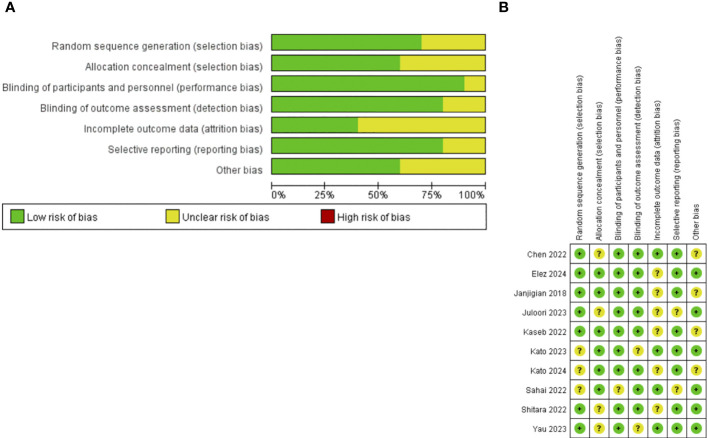
**(A)** Risk of bias graph. **(B)** Risk of bias summary.

Most studies were of good quality. Seven of the studies had a low risk of random sequence generation (selection bias), six had a low risk of allocation concealment (selection bias), nine had a low risk of blinding of participants and personnel (performance bias), eight had a low risk of blinding of outcome assessment (detection bias), four had a low risk of incomplete outcome data (attrition bias) and eight had selective reporting (reporting bias) (**Figure 2**).

### 3.3 Meta analysis of ORR

**Figure 3:**
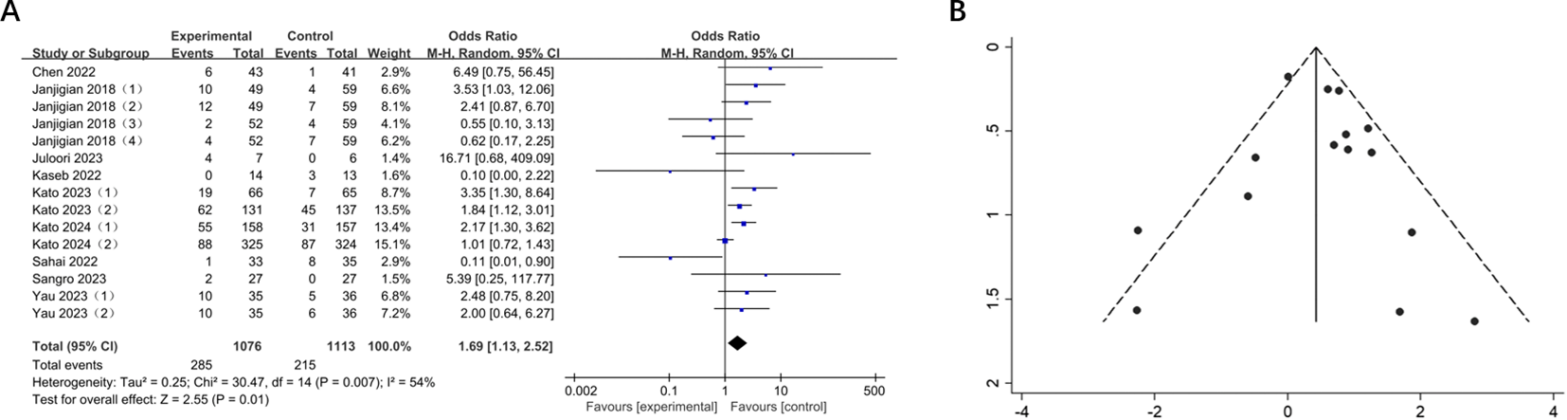
**(A)** Forest plot of ORR in patients with GI cancers treated with and without nivolumab in combination with ipilimumab. **(B)** Funnel plot of ORR in patients with GI cancers treated with and without nivolumab in combination with ipilimumab.

Nine trials reported ORR for 15 cohorts of patients with GI cancers including 2189 patients. There was a significant improvement in ORR in comparison with the control group (OR = 1.69, 95% CI: 1.13-2.52, P = 0.01), and the heterogeneity test showed moderate heterogeneity (I^2^ = 54%) (**Figure 3**). Therefore, the random effects model was adopted. Subsequent sensitivity analysis demonstrated that the removal of any single study did not exert a significant influence on the overall pooled results.

### 3.4 Meta analysis of mOS

**Figure 4:**
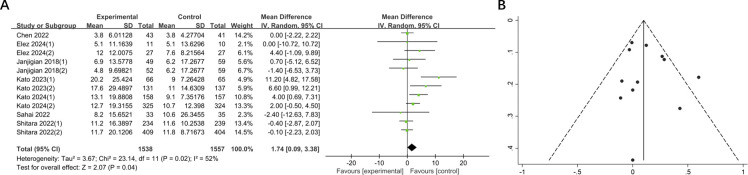
**(A)** Forest plot of mOS in patients with GI cancers treated with regimens containing and without nivolumab in combination with ipilimumab. **(B)** Funnel plot of mOS in patients with GI cancers treated with regimens containing and without nivolumab in combination with ipilimumab.

A total of 7 studies comprising 3095 patients reported mOS in 12 groups of patients with GI cancers. The mOS was significantly prolonged compared to the control (MD = 1.74, 95% CI: 0.09-3.38, P = 0.04). And heterogeneity test showed moderate heterogeneity (I^2^ = 52%) (**Figure 4**). Subsequent sensitivity analysis demonstrated that the removal of any single study did not exert a significant influence on the overall pooled results.

### 3.5 Meta analysis of mPFS

**Figure 5:**
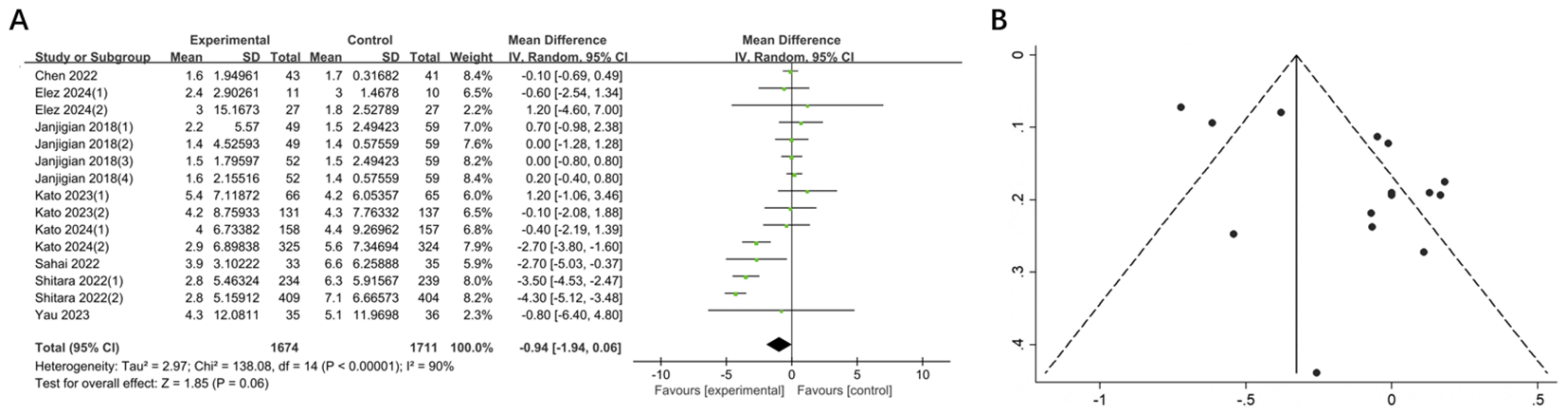
**(A)** Forest plot of mPFS in patients with GI cancers treated with regimens containing and without nivolumab in combination with ipilimumab. **(B)** Funnel plot of mPFS in patients with GI cancers treated with regimens containing and without nivolumab in combination with ipilimumab.

The mPFS in the experimental group was shorter than that in the control group, yet the difference was not statistically significant (MD = -0.94, 95% CI: -1.94-0.06, P = 0.06). Additionally, a high degree of heterogeneity was observed (I^2^ = 90%) (**Figure 5**). Sensitivity analysis demonstrated that no single study significantly influenced the high degree of heterogeneity.

### 3.6 Meta analysis of mDOR

**Figure 6:**
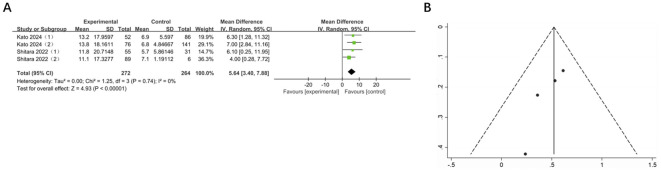
**(A)** Forest plot of mDOR in patients with GI cancers treated with regimens containing and without nivolumab in combination with ipilimumab. **(B)** Funnel plot of mDOR in patients with GI cancers treated with regimens containing and without nivolumab in combination with ipilimumab.

Two studies, involving 536 patients across 4 groups, reported mDOR for GI cancers. In comparison with the control group, mDOR was significantly extended in the experimental group (MD = 5.64, 95% CI: 3.40-7.88, P < 0.00001). There was no heterogeneity (I² = 0%) and publication bias (**Figure 6**).

### 3.7 Subgroup analysis

#### 3.7.1 Subgroup analysis of different tumors

Considering the moderate degree of heterogeneity in ORR and mOS observed in the data from the preceding studies, subgroup analyses were conducted to investigate potential differences in efficacy between experimental and control groups across diverse tumor types.

**Figure 7:**
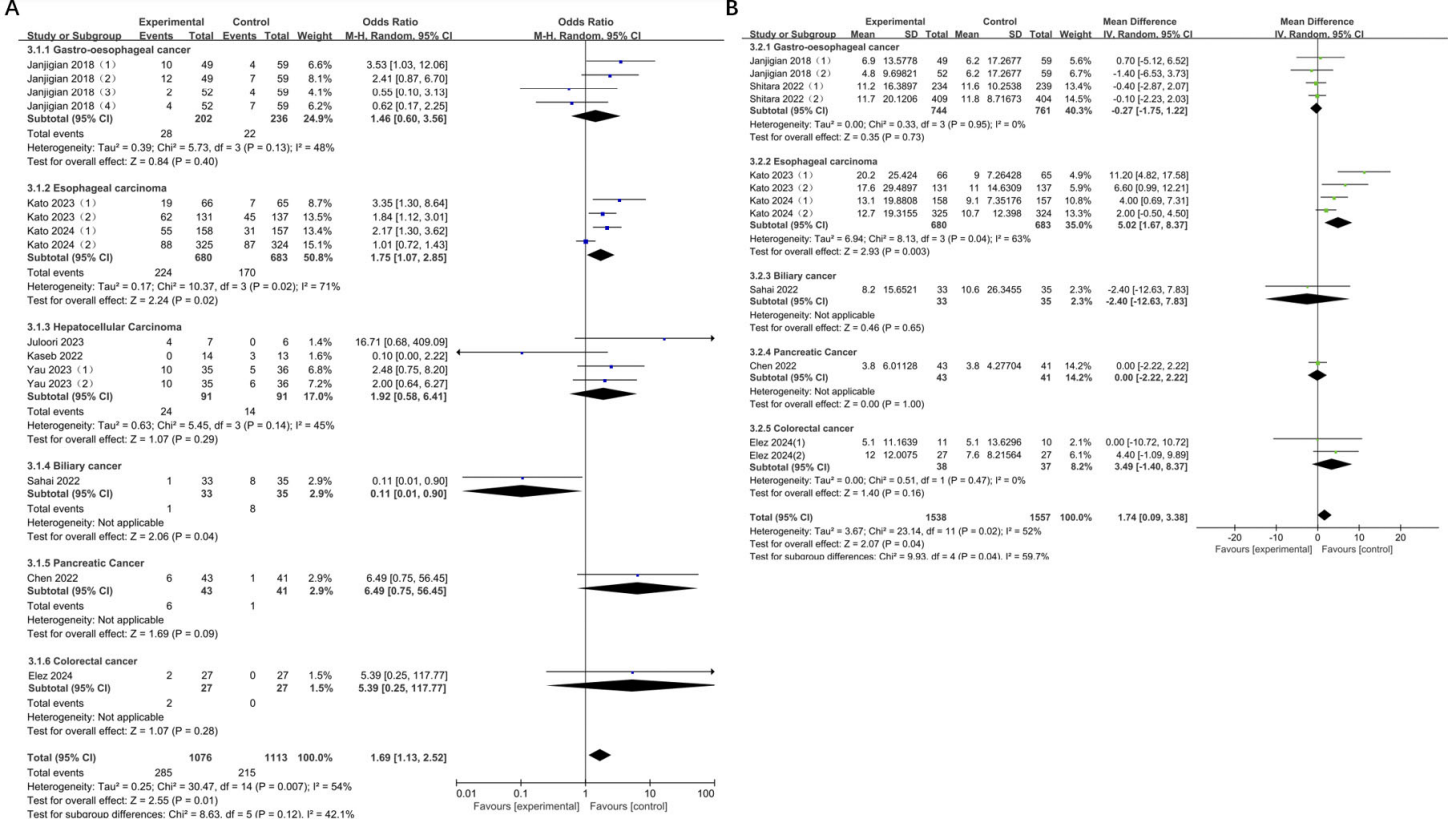
**(A)** Forest plot for subgroup analysis of ORR; **(B)** Forest plot for subgroup analysis of mOS.

The results showed a significant increase in ORR for esophageal cancer compared to the control group (OR = 1.75, 95% CI: 1.07-2.85, P = 0.02), with moderate heterogeneity (I² = 71%). The ORR for biliary cancer was significantly lower, which was statistically significant (OR = 0.11, 95% CI: 0.01-0.90, P = 0.04). There was a relatively significant increase in the objective remission rates of pancreatic cancer, colorectal cancer, gastroesophageal cancer and liver cancer, but none of them were statistically significant (pancreatic cancer: OR = 6.49, 95% CI: 0.75-56.45, P = 0.09; colorectal cancer: OR = 5.39, 95% CI: 0.25-117.77, P = 0.28; gastroesophageal cancer: OR = 1.46, 95% CI: 0.60-3.56, P = 0.40; hepatocellular carcinoma: OR = 1.92, 95% CI: 0.58-6.41, P = 0.29). However, only the heterogeneity in gastroesophageal cancer, esophageal cancer, and liver cancer were measurable, and no significant change was observed. (gastroesophageal cancer: I^2^ = 48%; esophageal cancer: I^2^ = 71%; hepatocellular carcinoma: I^2^ = 45%). A notable disparity in ORR was observed across different subgroups, as indicated by the subgroup differences (Chi² = 8.63, df = 5, P = 0.12, I² = 42.1%). Moreover, compared with the control group, the mOS of esophageal cancer was significantly prolonged, with a statistically significant difference (esophageal cancer: MD = 5.02, 95% CI: 1.67-8.37, P = 0.003). Conversely, no statistically significant difference was observed for the remaining tumors (**Figure 7**).

#### 3.7.2 Subgroup analysis of different dose ratios

The NIVO + IPI combination was approved as NIVO 1mg kg^−1^ + IPI 3mg kg^−1^ and NIVO 3mg kg^−1^ + IPI 1mg kg^−1^. Consequently, we extracted the valid data from six studies and reanalyzed the ORR, mOS, and mPFS of this combination based on the two-dose ratios.

**Figure 8:**
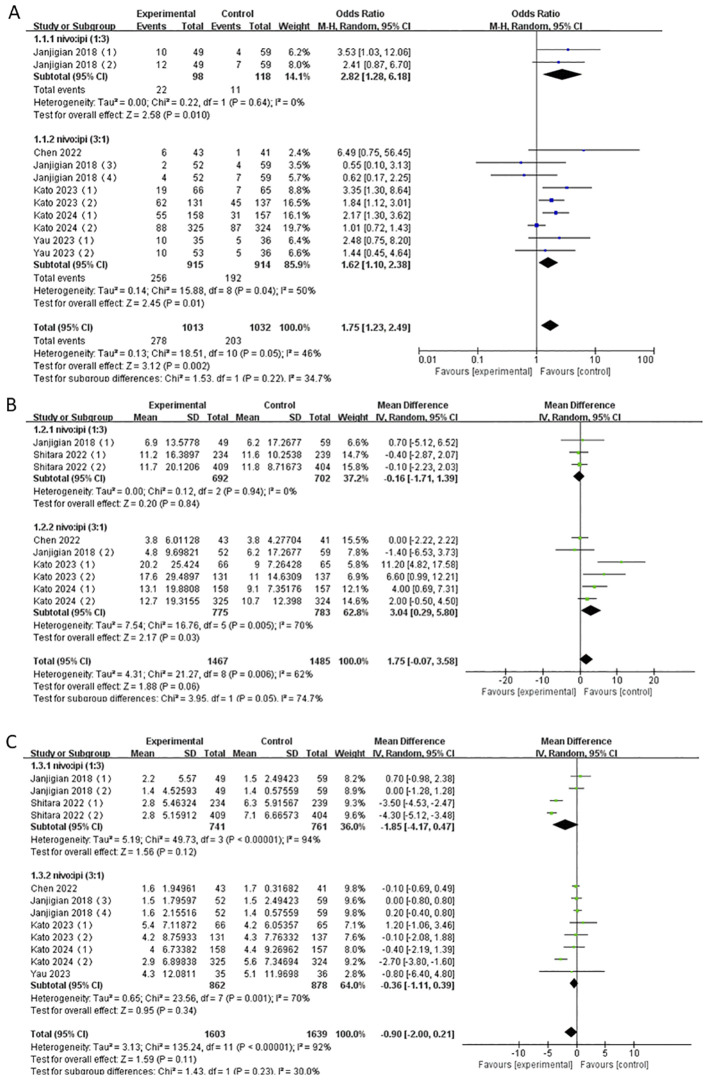
**(A)** Forest plot for subgroup analysis of ORR based on different doses. **(B)** Forest plot for subgroup analysis of mOS based on different doses. **(C)** Forest plot for subgroup analysis of mPFS based on different doses.

The results showed that the ORR of both the NIVO1 + IPI3 group and the NIVO3 + IPI1 group was higher than that of the control group (NIVO1 + IPI3: OR = 2.82, 95% CI: 1.28-6.18, P = 0.01; NIVO3 + IPI1: OR = 1.62, 95% CI: 1.10-2.38, P = 0.01), and there was low heterogeneity between the subgroups (I^2^ = 34.7%). In addition, the mOS and mPFS of the NIVO1 + IPI3 group and the NIVO3 + IPI1 group were not statistically significant (**Figure 8**).

### 3.8 Safety

**Figure 9:**
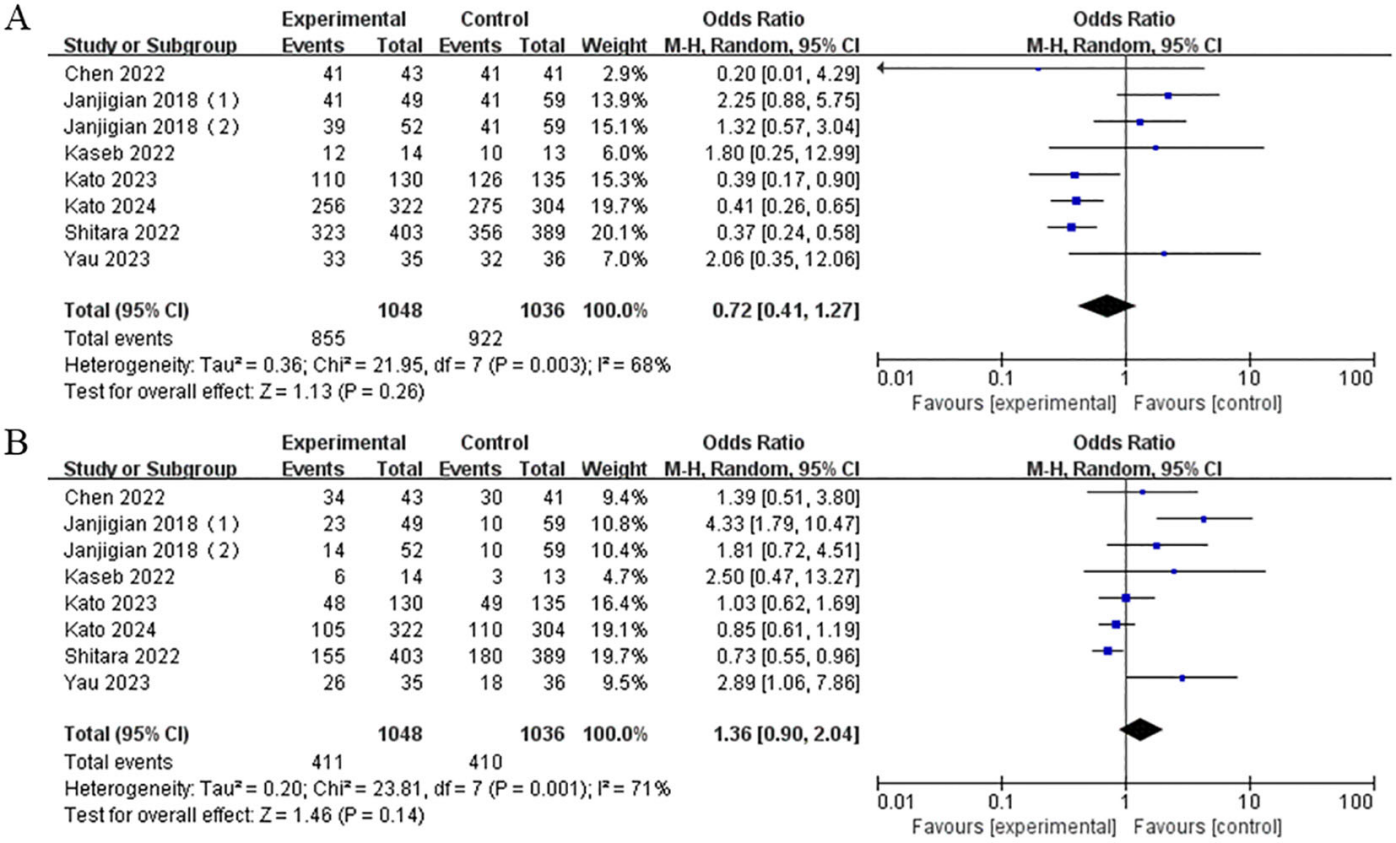
**(A)** Forest plot of any grade TRAEs in the combination of nivolumab and ipilimumab compared to the control group. **(B)** Forest plot of grade 3-4 TRAEs in the combination of nivolumab and ipilimumab compared to the control group.

The data on TRAEs were extracted from six studies. The results showed no statistically significant difference in the risk of TRAEs, either of any grade (OR = 0.72, 95% CI: 0.41-1.27, P = 0.26) or grades 3-4 (OR = 1.36, 95% CI: 0.90-2.04, P = 0.14), between the nivolumab-plus-ipilimumab treatment group and the control group (**Figure 9**).

## 4 Discussion

GI cancers are among the most lethal forms of cancer globally, accounting for a significant proportion of all tumor-related mortalities (2). As the number of elucidated molecular targets and targeted therapies continues to grow, the prospects and challenges associated with the exploration and identification of more effective immune-targeted therapy regimens become increasingly evident (38). To our knowledge, this is the first meta-analysis evaluating the efficacy and safety of nivolumab in combination with ipilimumab in GI cancers.

The results of the meta-analysis demonstrated that, in comparison with the control group, the combination of nivolumab and ipilimumab markedly enhanced the ORR and extended the mOS and mDOR in GI cancers. However, no statistically significant difference was observed in mPFS and the incidence of TRAEs of any grade or grade 3–4 reactions. Due to the moderate heterogeneity observed in ORR and mOS, subgroup analyses were performed based on tumor types. The results of subgroup analyses demonstrated a significant improvement in the ORR for esophageal cancer and a significant decline for biliary cancer. The remaining tumor types exhibited no statistically significant ORR, and the differences between the subgroups demonstrated low heterogeneity. Furthermore, only the mOS of esophageal cancer was significantly improved, and the heterogeneity between subgroups did not change significantly in comparison to the overall result. To gain a more comprehensive understanding of the clinical outcomes associated with this combination regimen, we conducted a subgroup analysis of ORR, mOS, and mPFS based on the dose ratio. The findings revealed that the ORR of both the NIVO1 + IPI3 group and the NIVO3 + IPI1 group was significantly higher than that of the control group, whereas no statistically significant differences were observed in mOS and mPFS.

Part of Our findings are in accordance with those of Parikh et al., who observed that the ORR and OS rate of nivolumab combined with ipilimumab in the treatment of advanced hepatocellular carcinoma were significantly superior to those of nivolumab monotherapy (30). Similarly, the survival benefits and acceptable tolerability observed in the NIVO + IPI therapy in the study by Kato et al. provided strong support for its use as the new standard first-line treatment for Japanese patients with advanced ESCC (10). The lack of a notable extension in mPFS observed in the study may be attributed to tumor heterogeneity and the mechanisms underlying immunotherapy. The effect of immunotherapy typically necessitates a specific period to activate the patient’s immune system, and the conventional PFS indicator is unable to fully capture the delayed effect of immunotherapy. As a result, some patients may experience transient disease progression before their immune system is fully activated, leading to PFS inadequately capturing the treatment’s efficacy promptly.

The results of a significant prolongation of ORR, mOS, and mDOR indicate that, although PFS was not significantly prolonged in some patients, the long-term effect may be more substantial and long-lasting once a response to immunotherapy is achieved. This may be attributed to the capacity of immunotherapy to enhance the activation of the patient’s anti-tumor immune system, establish long-term immune memory, and prevent rapid tumor recurrence. These findings have significant clinical implications, particularly for patients with early-stage disease who initially exhibit slow progression but eventually achieve a sustained response. It is recommended that patients who respond to immunotherapy be monitored over an extended period in clinical practice and that immunotherapy be considered as part of a long-term treatment plan, even in combination with other therapies to consolidate the treatment effect. Conversely, the heterogeneity of the ORR and mOS of this combination remains unresolved through subgroup analysis. Consequently, its clinical application should be exercised with caution.

The role of the tumor microenvironment in immunotherapy is of paramount importance. The TME of different types of GI cancers is highly heterogeneous, which has the potential to affect the efficacy of nivolumab and ipilimumab. In esophageal cancer, higher expression of PD-L1 and PD-L2 and greater T-cell infiltration have been observed to enhance tumor sensitivity to immunotherapy (39). In contrast, an immunosuppressive microenvironment (e.g. tumor-associated macrophages and tumor-infiltrating lymphocytes) has also been found to affect the prognosis of biliary tract cancer (40). Furthermore, the significant differences in the characteristic molecular targets of various GI cancers may also contribute to the observed heterogeneity in the efficacy of immune checkpoint inhibitors. The distinctive molecular characteristics of hepatocellular carcinoma are relatively concentrated and involve specific mutations or signaling pathways. These features include potential biomarkers such as interferon alpha (IFNα), alpha-fetoprotein (AFP), and transforming growth factor-beta (TGF-β), as well as the vascular endothelial growth factor (VEGF) pathway, mammalian target of rapamycin (mTOR) pathway, insulin-like growth factor 1 (IGF1) pathway and epidermal growth factor receptor (EGFR) pathway (41–43). Furthermore, numerous studies have demonstrated that multi-target tyrosine kinase inhibitors (TKIs)—such as sorafenib—can improve the survival rate of patients with advanced liver cancer to some extent (41, 44, 45). However, cholangiocarcinoma displays significant heterogeneity, with different biliary tract segments (including intrahepatic cholangiocarcinoma, extrahepatic cholangiocarcinoma, and gallbladder cancer) exhibiting distinct patterns of genetic mutations. For example, molecular abnormalities such as IDH1/2 mutations, FGFR2 fusions, and HER-2 overexpression are more common in some types of biliary tract cancer. However, these targets are only present in some patients, and the effect of targeted therapy is relatively limited. The use of anti-PD-1 or anti-CTLA-4 inhibitors in biliary tract cancer is uncommon and demonstrates limited efficacy (46). The disparate characteristics of TME and molecular targets are responsible for the varying responses observed in patients with GI cancers to the combination therapy of nivolumab and ipilimumab. Furthermore, a notable discrepancy was observed in the efficacy of nivolumab in combination with ipilimumab among patients with disparate PD-L1 expression levels within the same tumor. However, the literature included in the study provides only a limited range of efficacy data stratified by PD-L1 expression levels. This lack of data may contribute to the observed heterogeneity.

The combination of nivolumab and ipilimumab may represent a promising first-line treatment option for patients with GI cancers, particularly those with esophageal cancer. Nevertheless, for patients with biliary cancer, future clinical trials should investigate the potential of combining with other targeted or immune-enhancing therapies, given the likelihood of a poor response to immunotherapy. Furthermore, the findings of the study indicated that there was no notable enhancement in mPFS, thereby underscoring the necessity for greater emphasis on biomarker testing and dynamic efficacy assessment in clinical practice.

It is important to note that this study has limitations. Firstly, due to the paucity of data on TRAEs provided in the literature, our analysis yielded no statistically significant results compared to the control group. It is widely accepted that this combination regimen has the potential to induce adverse reactions. Consequently, further research is required to investigate the effects of this regimen on specific TRAEs, such as decreased appetite and fatigue. Secondly, although this study provided a comprehensive analysis of the combination of nivolumab and ipilimumab, the small sample size, particularly in subgroups such as biliary cancer, may have an impact on the reliability of the results. Furthermore, larger, multicenter randomized controlled trials are required to corroborate the findings of this study, particularly for tumor types exhibiting suboptimal efficacy.

## 5 Conclusion

In conclusion, this meta-analysis offers a comprehensive evaluation of the efficacy and safety of nivolumab combined with ipilimumab in patients with GI cancers, based on all available randomized controlled trials. The findings suggest that this combination therapy represents a promising and effective option for managing GI cancers. However, caution is warranted in interpreting these results due to significant variability arising from the molecular heterogeneity of different GI cancer types.
